# Ongoing recruitment crisis In Norwegian general practice

**DOI:** 10.1080/02813432.2018.1462294

**Published:** 2018-04-25

**Authors:** Svein R. Kjosavik

**Affiliations:** National Editor of Scandinavian Journal ofPrimary Health Care; Stavanger University Hospital, Stavanger, Norway;; Faculty of Health Sciences, University of Stavanger, Stavanger, Norway

In Norway, a National Regular General Practitioner Scheme started in 2001, giving all inhabitants the right to choose a GP as their regular doctor [1]. The scheme is managed by the Norwegian Health Economics Administration, but the municipalities are responsible for entering into agreements with each GP. About 99% of the population have their own GP, and overall satisfaction is high [2].

A new health care reform called the Care Coordination Reform has gradually been implemented since 2012 [3]. This reform aimed to improve the collaboration and coordination between primary and secondary health care. The municipalities were supposed to take on the responsibility for more patients, avoid referrals to hospital, and receive patients from the hospital at an earlier stage. Each GP got increased responsibility for the course of treatment for each patient, but was supposed to be responsible for fewer patients. The Minister of Health who initiated the reform, Bjarne Håkon Hanssen, announced a need for 2200 new GPs, but from 2011 to 2016 the number increased with less than 500. During the same period, the population increased with about 340,000. Consequently, GPs have many new tasks to take care of although the promised increase in the number of primary care physicians has failed [4].

The deficit of GPs has repeatedly been problematized, but little has happened until a group of GPs in Trondheim (in the county of Trøndelag) started a “grass root” campaign for change last summer called *the Trønder rebellion.* During the fall of 2017, the uprising spread across most of the country. The message is that the increased transfer of tasks from secondary to primary health care is appropriate, but primary health care needs to be strengthened and structured in a proper way to take care of the increased workload. How this will be achieved leaves many crucial questions.

It seems reasonable to follow the plan from the former Minister of health. In the short term, the framework conditions need improvement to attract more doctors to general practice, and the number of patients on the GP’s list need to be reduced. Salary systems should be more flexible and context sensitive. In addition, there is a need to ensure enough staffing with qualified personnel at the doctor's offices.

In a broader view, an important challenge is the education of doctors. Today, 3875 are studying medicine in Norway. According to the Norwegian State Educational Loan Fund, they also support 3279 medical students abroad. Accordingly, more than 45% of Norwegian medical students get their education outside of a Norwegian context. Many of these receive little training in general practice, compared to the Nordic medical schools. The Norwegian Medical Association recommends that the level of doctors educated abroad should be less than 15%.

However, the challenge is not only to educate enough physicians with a solid foundation in general practice, but also to make general practice an attractive career choice for young doctors. *The Trønder rebellion* has addressed an increasing tendency to professional exhaustion and demoralization among listed GPs in Norway, along with a significant recruitment crisis. The result might be a downward spiral. A survey conducted by The Royal College of General Practitioners (RCGP) and Medical Schools Council (MSC) among medical students in the UK, showed that students are strongly influenced by their peers, and their perception of general practice formed during their studies [5]. A Norwegian study from 2012 showed significant associations between medical schools and the proportion of physicians choosing a career as GPs after graduation [6]. Contextual factors and local role models influence recruitment to general practice [7].

Formally, general practice has become a core discipline in all the four Norwegian medical schools. So far, these adjustments in the curricula have not given the desired increase in the number of doctors who choose a career in general practice. According to Statistics Norway, 4.8 new physicians were employed in hospitals per every new GP in the period from 1990–2010, while the figure was 6.5 for the period from 2011 to 2016. The Norwegian Medical Association has emphasized the importance of dimensioning education according to future needs, for instance by establishing a medical curriculum at a fifth university. In addition, The Norwegian Parliament recently asked the government to consider increasing the domestic number of medical students, and to strengthen the training in primary health care.

However, even more needs to be done to increase the domestic proportion of students to 85% or more. In order to ensure the students adequate access to patients during their education, it will be necessary to develop more decentralized training, utilizing the capacity of smaller hospitals and rural physicians. Canada and Australia have a lot of experience with such decentralized solutions, resulting in better recruitment to generalist medicine, particularly in rural areas [8,9]. Digital tools have made it possible to achieve this while maintaining high quality in teaching. In Norway, Tromsø has already started a rural track, and Trondheim will start one in 2018.

Until recently, Norwegian authorities hardly listened to the many repeated and serious warnings from general practice. I hope that the ongoing grass root campaign will bring the debate to a tipping point and lead to change. It is time the authorities make decisions that provide good and sustainable solutions, both in the short and the long term.
